# Similar adverse outcome rates with high or low oxygen saturation targets in an area with low background mortality

**DOI:** 10.3389/fped.2023.1235877

**Published:** 2023-10-24

**Authors:** Nina Willgerodt, Christoph Bührer, Rainer Rossi, Thomas Kühn, Mario Rüdiger, Stefan Avenarius, Ralf Böttger, Dirk M. Olbertz, Hans Proquitte, Hans-Jörg Bittrich, Roland Haase, Matthias Fröhlich, Sybille Höhne, Ulrich H. Thome

**Affiliations:** ^1^ Division of Neonatology, University Hospital for Children, Leipzig, Germany; ^2^ Department of Neonatology, Charité—Universitätsmedizin Berlin, Berlin, Germany; ^3^ Division of Neonatology, Vivantes Hospital Neukölln, Berlin, Germany; ^4^ Division of Neonatology and Pediatric Intensive Care Medicine, Klinik für Kinderheilkunde, Medical Faculty, TU Dresden, Dresden, Germany; ^5^ Division of Neonatology, University Hospital for Children, Magdeburg, Germany; ^6^ Division of Neonatology, Hospital Südstadt, Rostock, Germany; ^7^ Division of Neonatology, University Hospital for Children, Jena, Germany; ^8^ Division of Neonatology, Helios Hospital, Erfurt, Germany; ^9^ Division of Neonatology, University Hospital for Children, Halle (Saale), Germany; ^10^ Division of Neonatology, St. Elisabeth and St. Barbara Hospital, Halle (Saale), Germany

**Keywords:** preterm infant, oxygen saturation, oxygen dilemma, mortality, bronchopulmonary dysplasia, retinopathy of prematurity, necrotizing enterocolitis, intraventricular hemorrhage

## Abstract

**Background:**

Randomized controlled trials have indicated reduced mortality rates in very preterm infants assigned to high compared to low oxygen saturation (SpO_2_) target levels, accompanied by higher rates of retinopathy of prematurity and bronchopulmonary dysplasia. However, the benefit-to-harm ratio may depend on the local background mortality risk. We therefore aimed to quantify the risk–benefit ratios of different SpO_2_ target ranges in 10 tertiary newborn intensive care units (NICUs) in East Germany.

**Methods:**

In a retrospective multicenter study, 1,399 infants born between 2008 and 2012 at a gestational age between 24 0/7 and 27 6/7 weeks and with a birthweight below 1,250 g were grouped according to the hospital's target SpO_2_ range [high oxygen saturation group (HOSG) above 90%], low oxygen saturation group (LOSG) below 90%] and the compliance of units with their target SpO_2_ range. The association between neonatal morbidities, neurodevelopmental outcomes, selected treatment strategies, and target SpO_2_ ranges was calculated using chi-squared and Mann Whitney *U* tests.

**Results:**

Nine of the ten participating NICUs met their SpO_2_ target ranges. Five units were considered as HOSG, and five units were considered as LOSG. Necrotizing enterocolitis and intraventricular hemorrhage grade ≥ 2 occurred significantly more frequently in the HOSG than in the LOSG (8.4% vs. 5.1%, *p* = 0.02; and 26.6% vs. 17.7%, *p* < 0.001). No significant differences in the mortality rate and the rate of retinopathy of prematurity were found.

**Conclusion:**

In our patient population, a lower SpO_2_ target range was not associated with increased safety risks in extremely preterm infants. We cannot be sure that our outcome differences are associated with differences in oxygen saturations due to the retrospective study design and the differences in site practices.

## 1. Introduction

Premature infants, particularly those born less than 28 weeks gestation, often require respiratory support, including oxygen supplementation after birth. Although lifesaving, oxygen supplementation and ventilation pose significant health risks. Oxygen supply and oxygen toxicity certainly play an important part in developing some diseases typical for preterm infants (1–3). Furthermore, preterm small-for-gestational-age (SGA) infants have even higher risk of disease and an increased mortality rate than appropriate-for-gestational-age (AGA) infants (4–6). Although survival of preterm infants with low gestational age has increased in recent years, disability rates for these infants remain high (7, 8). Due to their immature organ systems and insufficiently developed antioxidant defenses, the risk of premature infants developing various diseases such as bronchopulmonary dysplasia (BPD), retinopathy of prematurity (ROP), or necrotizing enterocolitis (NEC) is modified by treatment with oxygen, hypoxia or hyperoxia.

BPD and ROP are, among other factors, related to hyperoxygenation and oxygen toxicity (9). Randomized trials confirmed that liberal oxygen therapy increases the incidence of both BPD and ROP (10–12). Thus, restricted oxygen therapy has the potential to reduce BPD and ROP.

On the other hand, NEC, a life-threatening gastrointestinal disease with a high mortality rate, was found to be increased when oxygen therapy was restricted (13).

Intraventricular hemorrhage (IVH) can lead to brain injury and severe lifelong disabilities (14). A multifactorial pathogenesis is assumed. Risk factors include reduced or varying cerebral blood flow, hypoxia, hypercapnia, patent ductus arteriosus, or severe respiratory distress syndrome (15). A clear relation to oxygen saturation targeting has not been established.

Five randomized trials of target oxygen saturation in preterm infants were conducted from 2005 to 2010: BOOST (Benefits of Oxygen Saturation Targeting) II, BOOST II UK, BOOST NZ, SUPPORT (Surfactant Positive Pressure and Oxygenation Randomized Trial), and COT (Canadian Oxygen Trial) (16–18). The data of 4,965 infants were combined as part of the NeOProM (Neonatal Oxygen Prospective Meta-Analysis) collaboration (10, 13, 19). In all trials, infants with a gestational age of less than 28 weeks were randomly assigned to a higher oxygen saturation target of 91%–95% or a lower oxygen saturation target of 85%–89% immediately after birth or shortly thereafter (16–18). Both the SUPPORT trial and the BOOST trials observed a lower rate of severe ROP but, at the same time, a significantly increased mortality rate in the lower oxygen saturation target groups (16, 18). Of note, an increased incidence of NEC was decisive for the increased mortality in the BOOST trials (16). The most recent of the three studies, COT, observed neither a significant reduction in the ROP rate nor a significant difference in the mortality rate between the two saturation ranges (17). The rates of BPD, however, were not different between the groups in all three trials (16–18).

In these trials, the higher mortality associated with lower oxygen saturation targets was superimposed on a rather high background mortality and a rather high incidence of NEC: the cumulative mortality rate before discharge from the hospital in the NeOProM trials was 19% in the lower target group and 16% in the higher target group, and the cumulative NEC rate was 9% in the lower target group and 7% in the higher target group (13). In comparison, we found a mortality rate of 12.8% in the low oxygen saturation group (LOSG) and 14.7% in the high oxygen saturation group (HOSG), and a NEC rate of 5.1% in the LOSG and 8.4% in the HOSG. The gestational age as a possible influencing factor on the mortality rate in our study was similar to NeOProM, with a median of 26 weeks and an interquartile range of 25–27 weeks. Thus, the risk–benefit ratios found in these trials may not apply to centers with lower rates of background mortality and NEC (10, 20). It is thus improbable that one uniform oxygen saturation range will result in the best possible outcomes in all centers (21). Therefore, oxygen therapy may better be individualized among centers and individual patients according to various parameters such as prenatal complications, gestational age, mode of delivery, or sex (21).

In this retrospective, multicenter study at 10 German tertiary care NICUs, we examined how different SpO_2_ targets were associated with outcome data in NICU settings with much lower mortalities and NEC incidences than in the NeOProM trials. We hypothesized that the observed benefits of high oxygen targets may be diminished in the participating hospitals, possibly even favoring lower targets. Therefore, we also provide insights into how different background risks in different populations influence risk/benefit ratios (20).

## 2. Methods

The study was approved by the ethics committee of the Medical Faculty of the University of Leipzig. A retrospective data survey was performed, which included 1,399 preterm infants with a birth weight of less than 1,250 g and a gestational age of 24 weeks or more but less than 28 weeks. The infants were born in the period from January 1, 2008, to December 31, 2012, in one of the 10 participating hospitals and nursed in the respective NICUs, all located in the eastern parts of Germany. These were the Charité–Universitätsmedizin Berlin, Vivantes Hospital Berlin-Neukölln, University Medical Center Leipzig, University Hospital Carl Gustav Carus Dresden, University Medical Center Magdeburg, Hospital Südstadt Rostock, University Medical Center Jena, Helios Hospital Erfurt, University Medical Center Halle (Saale), and St. Elisabeth Hospital Halle (Saale). Only infants born in the hospitals’ delivery wards (inborn) were included.

Exclusion criteria were death in the delivery room and severe malformations. See the Appendix for a list of exclusion diagnoses. Data were extracted from quality control databases and original patient records and stored in a pseudonymized database for analysis.

Participating NICUs were divided into two groups: NICUs with relatively high and NICUs with low SpO_2_ target ranges. A strict separation was, however, not possible due to the retrospective study design. NICUs aiming above 90% were assigned to the HOSG, and NICUs aiming below 90% were assigned to the LOSG. The classification was based on the studies of the NeOProM Collaboration, which classified oxygen saturations of 91%–95% as high and oxygen saturations of 85%–89% as low (10, 19).

NICUs provided their predefined target ranges for SpO_2_ during the study period to determine target ranges. Intercurrent changes in the target ranges and special guidelines for different age groups were also considered. Infants of one year group were defined as those born within the same year (2008, 2009, 2010, 2011, or 2012) at the same hospital. Adherence of units to their target ranges was tested by a more detailed data acquisition, including all SpO_2_ and oxygen fraction (FiO_2_) values in five randomly selected patients per year group from each participating NICU.

For these 25 infants per hospital, hourly SpO_2_ and set FiO_2_ values were included in the analysis for 13–60 h of life. For the first 12 h of life, only the FiO_2_ values were analyzed to determine the maximum FiO_2_ and test whether the NICUs with a higher saturation target used higher FiO_2_. Subsequently, saturation values above the NICUs target range at an FiO_2_ of 0.21 and saturations below the NICUs target range at an FiO_2_ of 1.0 were excluded from this analysis, as these were beyond the influence of medical treatment.

From the saturations of 13–60 h of life, the median for each selected infant and the median of the medians of all infants of each NICU were calculated. If this median was within the saturation target range of the respective NICU, the saturation target corridor specified by the NICU was used for the group assignment. If the median was outside the target corridor, the group assignment was made according to the actual range of SpO_2_ medians observed.

The neurological outcome at the age of 24 months and the occurrence of disabilities were estimated by follow-up examinations using the Bayley scales for infant development II or III (22). The measurements were recorded as a mental developmental index (MDI). For the conversion from Bayley III Cognitive Composite (CC) to Bayley II MDI, we used the following equation: MDI=−35.065+(1.234)×CC (22). For NICUs only providing the cognitive development age in months, a conversion into index values using raw value equivalents was necessary. The conversion tables from the Bayles Scales of Infant Development 2nd Edition BSID II manual by N. Bayley were used (23). PDI values were not determined in some centers, preventing a meaningful analysis.

BPD was graded according to the definition of Jobe and Bancalari (24), ROP was graded according to the International Classification of Retinopathy of Prematurity of 2005 (25), IVH was graded according to Papile et al. (26), and NEC was graded according to Bell et al. (27).

For the statistical analysis, patients were first assigned to the HOSG or LOSG according to the group assignment of their NICU as described above. The influence of target SpO_2_ and other variables on neonatal mortality was calculated using binary logistic regression. Odds ratios in the regression analysis were not adjusted.

The association between the occurrence of typical preterm birth disorders, neurodevelopmental outcomes, selected treatment strategies, and target SpO_2_ was calculated for nominal characteristics using a chi-squared test or Fisher's exact test as applicable. Analysis of metric, non-normally distributed variables against SpO_2_ was performed using the Mann–Whitney *U*-test. Statistical significance is a two-sided *p*-value <0.05 without adjustment for multiple testing. Nominal data were described with frequency and relative frequency. Numerical data were described as medians and interquartile ranges or as means and standard deviations. No adjustments were applied for multiple testing. IBM SPSS Statistics software (Version 28.0) was used throughout.

## 3. Results

**Figure 1:**
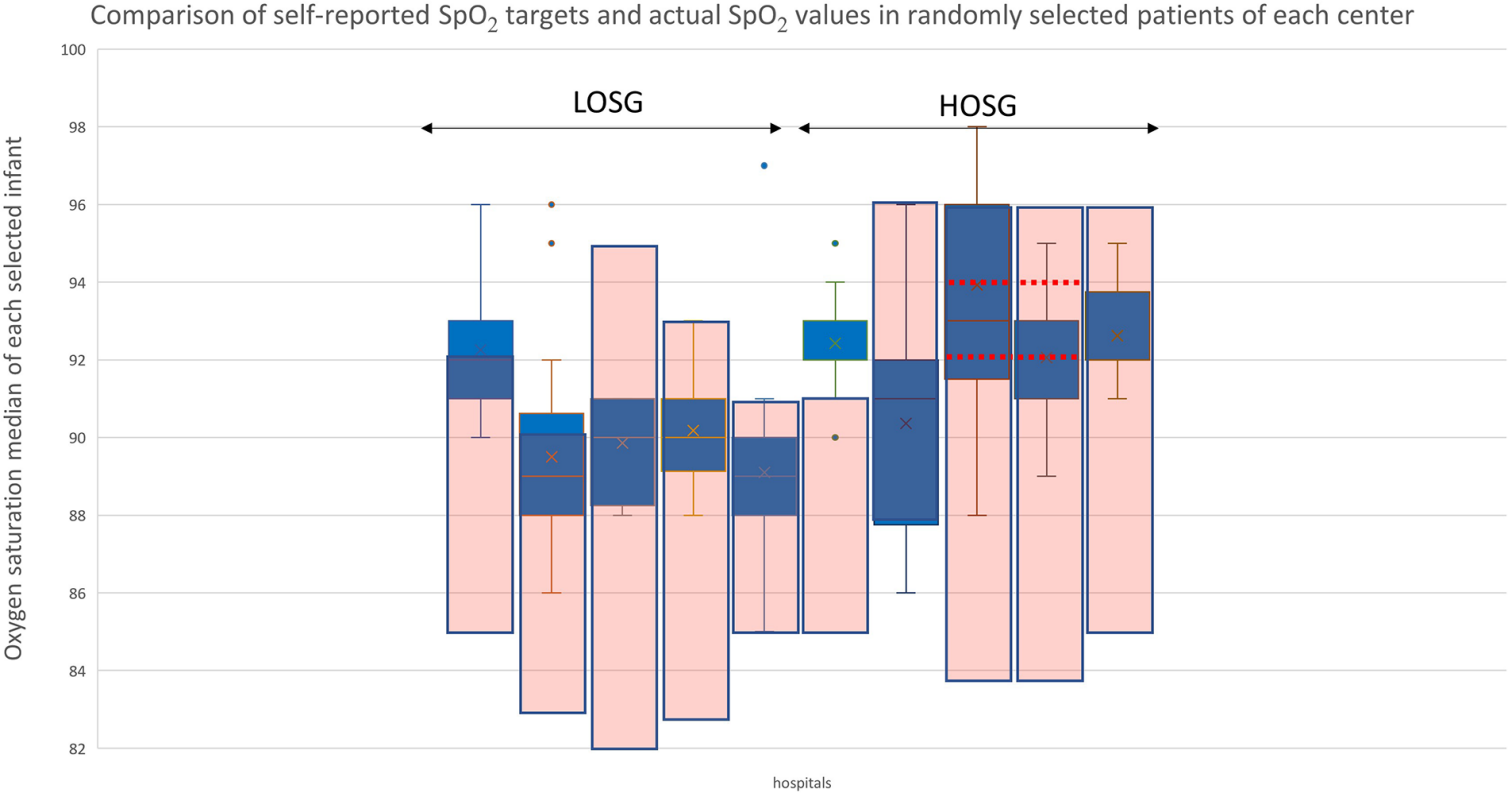
Comparison of self-reported SpO_2_ targets and actual SpO_2_ values in randomly selected patients of each center. The blue box-and-whisker plots represent the minimum, maximum, median, and quartile of each hospital's sample. The pink boxes show the hospital's SpO_2_ target ranges. Some hospitals also specified “optimal target areas” within their target range, which are indicated by horizontal red dashed lines.

During the study period, 1,399 preterm infants fulfilled the inclusion criteria. The detailed analysis of randomly selected patients revealed that 9 of the 10 participating NICUs met their SpO_2_ target ranges. By analyzing the measured oxygen saturations, five NICUs were assigned to the HOSG and five NICUs were assigned to the LOSG. Only one NICU with a reportedly low target range of 85%–91% had to be reassigned to the HOSG due to a median of 92% and a range of the medians from 90% to 95% (Figure 1). Of the 1,399 included patients, *n* = 1,019 were cared for in a low-SpO_2_-target NICU and *n* = 380 were cared for in a high-SpO_2_-target NICU (Table 1).

**Table 1:** Baseline characteristics: data are presented as median (MD) and interquartile range (IQR), mean (MV) and standard deviation (SD), or a percentage of the respective subgroup.

		LOSG (*n* = 1,019)	HOSG (*n* = 380)	*p*-value
Gestational age, weeks, MV ± SD		26 ± 1	26 ± 1	<0.05
Sex, *n* (%)	Male	563 (55.3)	196 (51.6)	0.22
Birth weight, g, MV ± SD		797 ± 197	812 ± 201	0.21
Multiple births, *n* (%)		300 (29.4)	74 (19.5)	<0.05
SGA, *n* (%)		244 (23.9)	93 (24.5)	0.84
Chorioamnionitis, *n* (%)		158 (15.5)	54 (14.2)	0.55
Administration of a surfactant, *n* (%)		461 (45.2)	208 (54.7)	<0.05
Apgar score at 5 min, MD (IQR)		7 (6–8)	7 (6–8)	0.69
Umbilical artery pH value, MD (IQR)		7.32 (7.26–7.36)	7.33 (7.27–7.38)	<0.05
Antenatal steroid use, *n* (%)		853 (83.7)	259 (68.2)	<0.05
		LOSG (*n* = 1,014)^a^	HOSG (*n* = 377)^a^	
Mode of delivery, *n* (%)	Cesarean section	903 (89.1)	335 (88.9)	0.92

^a^ Lower number of infants due to missing data for eight infants.

Regarding the baseline characteristics, the infants in our saturation groups differed significantly in their gestational age, the proportion of multiple births, their umbilical artery pH value, the receipt of antenatal steroids, and the use of surfactant (Table 1).

Several variables had a significant association with the mortality rate: a 1-week increase in gestational age reduced the mortality rate by 27.2%, *p* < 0.01. Girls were 42.3% less likely to die than boys, *p* < 0.01. If birth weight increased by 50 g, the relative mortality rate decreased by 10.0%, *p* < 0.05. If the Apgar score at 5 min increased by one unit, the relative mortality rate decreased by 22.8%, *p* < 0.001. The SpO_2_ target group assignment, however, did not affect mortality (see Table 1 in the Supplementary material).

The use of certain treatments differed significantly between the hospitals in the two saturation target groups. Infants in the HOSG hospitals were significantly more often treated with systemic and inhaled steroids than in the LOSG hospitals. Ventilation with NO, high-frequency oscillatory ventilation (HFOV), and ibuprofen administration were significantly more frequently administered in the LOSG hospitals (Table 2).

**Table 2:** Treatment strategies: data are presented as a percentage of the respective subgroup.

	LOSG (*n* = 1,019)	HOSG (*n* = 380)	*p*-value
Postnatal systemic steroids, *n* (%)	226 (22.2)	121 (31.8)	<0.001
Ventilation with NO, *n* (%)	88 (8.6)	19 (5.0)	0.02
HFOV ventilation, *n* (%)	302 (29.6)	83 (21.8)	0.004
Administration of ibuprofen, *n* (%)	348 (34.2)	85 (22.4)	<0.001
	LOSG (*n* = 1,006)^a^	HOSG (*n* = 380)	
Inhaled steroid, *n* (%)	79 (7.9)	58 (15.3)	<0.001

^a^ Lower number of infants due to missing data for 13 infants.

There were several significant differences according to the SpO_2_ target group. NEC stage 2 or higher was diagnosed significantly more frequently in the HOSG. IVH grade 2 or higher was significantly more common in the HOSG (Table 3). The ROP and BPD rates and the MDI were not different between the SpO_2_ target groups (Table 3).

**Table 3:** Outcome data: data are presented as a percentage of the respective subgroup.

		LOSG (*n* = 1,019)	HOSG (*n* = 380)	*p*-value
Deceased, *n* (%)		130 (12.8)	56 (14.7)	0.33
NEC, *n* (%)		52 (5.1)	32 (8.4)	0.02
IVH ≥ 2, *n* (%)		180 (17.7)	101 (26.6)	<0.001
		LOSG (*n* = 925)^a^	HOSG (*n* = 338)^a^	
BPD, *n* (%)	No BPD	326 (35.2)	103 (30.5)	0.13
	Mild	415 (44.9)	150 (44.4)	
	Moderate	143 (15.5)	62 (18.3)	
	Severe	41 (4.4)	23 (6.8)	
		LOSG (*n* = 862)^b^	HOSG (*n* = 320)^b^	
ROP, *n* (%)	No ROP	462 (53.6)	167 (52.2)	0.43
	Stage 1	145 (16.8)	54 (16.9)	
	Stage 2	146 (16.9)	54 (16.9)	
	Stage 3	104 (12.1)	40 (12.5)	
	Stage 4	2 (0.2)	4 (1.3)	
	Stage 5	3 (0.3)	1 (0.3)	
		LOSG (*n* = 569)^c^	HOSG (*n* = 249)^c^	
MDI, *n* (%)	<70	107 (18.8)	44 (17.7)	0.93
	70–85	118 (20.7)	52 (20.9)	
	>85	344 (60.5)	153 (61.4)	

^a^ Lower number of infants due to death of 135 infants before week 36 or missing data for 1 infant.

^b^ Lower number of infants due to death of 139 infants before screening in week 33 or missing data for 78 infants.

^c^ Lower number of infants due to death of 186 infants or missing data for 395 infants.

The highest FiO_2_ during the first 12 h of life and the duration of oxygen administration were significantly higher in the HOSG than in the LOSG. The duration of continuous positive airway pressure (CPAP) support was significantly higher in the LOSG than in the HOSG. The total duration of respiratory support was significantly higher in the LOSG compared to the HOSG. Gestational age at discharge and invasive ventilation duration did not differ significantly between the groups (Table 4).

**Table 4:** Duration of support measures: data are presented as median (MD) and interquartile range (IQR) and obtained by the Mann–Whitney *U*-test.

	LOSG (*n* = 1,019)	HOSG (*n* = 380)	*p*-value
Duration of invasive ventilation, days, MD (IQR)	5 (1–19)	6 (1–21)	0.19
Duration of oxygen administration, days, MD (IQR)	38 (5–64)	41 (18–70)	0.004^a^
Duration of CPAP administration, days, MD (IQR)	26 (10–42)	16 (5–31)	<0.001^a^
Total duration of respiratory support, days, MD (IQR)	37 (20–57)	31 (12–45)	<0.001^a^
Postmenstrual age at hospital discharge, weeks, MD (IQR)	37 (35–40)	37 (34–39)	0.21
	LOSG (*n* = 998)^b^	HOSG (*n* = 310)^b^	
Highest oxygen fraction of the first 12 h of life, MD (IQR)	0.35 (0.25–0.5)	0.4 (0.3–0.6)	<0.001^a^

^a^ Correlation is significant at the 0.05 level (two-tailed).

^b^ Lower number of infants due to missing data for 91 infants.

## 4. Discussion

Although oxygen supplementation has been an indispensable method in the care of preterm infants since the 1940s (10) and pulse oximetry has been used to guide oxygen therapy since the 1980s, there is considerable uncertainty about which SpO_2_ target is the safest for preterm infants. Oxygen is a double-edged sword, which results in the so-called “oxygen dilemma” (28): lower oxygen saturations by restrictive oxygen application may affect neurodevelopment or lead to death, while higher oxygen saturations by more liberal oxygen application may increase the number of cases with BPD or ROP (10). Different oxygen dosing associated with both high and low SpO_2_ targets pose risks for complications in preterm infants. Since risks must be balanced against each other, different health systems with different complication rates may have different optimal ranges for oxygen therapy (20). For these reasons, we have evaluated the risk profiles of high and low SpO_2_ targets in tertiary NICUs in the eastern part of Germany, which has comparatively few but large perinatal centers with a relatively low infant mortality rate (29).

Owing to our study design, differences in outcomes discussed below may not be related to the differences in oxygen saturation targets because baseline differences in infants and several treatment strategies differed between the participating NICUs. The expected mortality disadvantage in the LOSG was not found, even though the infants in the LOSG were slightly more lightweight and had a higher proportion of males (here, we refer again to our Supplementary material for a binary logistic regression about how some variables changed the risk of death during the course in our data). A type II error originating from an insufficient sample size cannot be fully excluded but appears to be unlikely since there was not even a trend favoring the HOSG. Thus, there was no indication that collecting a much higher number of cases would have produced such a difference with statistical significance. The rates of NEC and IVH were significantly higher in the HOSG. As NEC is a disease with a high mortality rate (30), a higher rate of NEC and a tendency toward higher mortality in the same SpO_2_ target group are plausible combinations. Furthermore, we did not find any significant differences in the rates of ROP and BPD between the SpO_2_ target groups, although BPD, like mortality, tended to be somewhat higher in the HOSG.

As previously hypothesized, our results do not align in all outcome parameters with the five previous NeOProM randomized trials comparing higher with lower SpO_2_ targets (Table 5) (13, 19). Despite common planning, some results differed between these trials, demonstrating the need for further research. The SUPPORT and the BOOST trials observed a higher mortality rate in their lower SpO_2_ target range group, as well as more cases of severe NEC in the BOOST trials, which appeared to be interrelated, while the incidences of ROP and BPD were lower (16, 18). The COT trial, however, found neither a significant reduction in the ROP rate nor a significant difference in the mortality rate between the two SpO_2_ target ranges (17). It can thus be seen that the differences in outcomes between the saturation groups decreased from the oldest (SUPPORT) to the most recent trial (COT) (for more information, we refer to the table “Comparison of the incidences in the NeOProM trials and our study” in the Supplementary material). Because of the diverging risks and benefits among the SpO_2_ target groups, it has been hypothesized that the risk–benefit ratio of SpO_2_ targets at different NICUs may depend on the specific risks of background mortality and NEC (20).

**Table 5:** Comparison of the incidences in the NeOProM trials and our study.

	NeOProM		Our results	
	No. of infants with event/total no. (%)		No. of infants with event/total no. (%)	
	Lower SpO_2_ target	Higher SpO_2_ target	Lower SpO_2_ target	Higher SpO_2_ target
Death before discharge from the hospital	460/2,478 (19)^a^	397/2,481 (16)^a^	130/1,019 (12.8)	56/380 (14.7)
Treated retinopathy of prematurity before corrected age of 18–24 months	220/2,020 (11)^a^	308/2,065 (15)^a^	109/862 (12.6)^b^	45/320 (14.1)^b^
Severe necrotizing enterocolitis	227/2,464 (9)^a^	170/2,465 (7)^a^	52/1,019 (5.1)^a^,^c^	32/380 (8.4)^a^,^c^
Supplemental oxygen at postmenstrual age of 36 weeks	459/1,846 (25)^a^	578/1,910 (30)^a^	184/925 (19.9)	85/338 (25.1)

^a^ Correlation is significant at the 0.05 level (two-tailed).

^b^ Defined as ROP ≥ stage 3.

^c^ Defined as NEC ≥ stage 2.

In this retrospective multicenter study, we analyzed the risks and benefits of different SpO_2_ targets in Eastern Germany. This area is characterized by a well-developed centralization of tertiary neonatal care and a comparatively low infant mortality rate (29). Indeed, the mortality in this study (13.3% overall) was lower than in the NeOProM trials (17.3% deaths before discharge overall) (13) (Table 5). Compared to the NeOProM trials, we found a similar trend in the incidence of BPD favoring the LOSG, which is corroborated by higher postnatal steroid use in the HOSG, probably because steroids were used to alleviate BPD. In contrast to the NeOProM trials, the rates of NEC and IVH were significantly higher in our HOSG than in LOSG. The rates of mortality and ROP were not different (17, 18). As expected, the duration of oxygen administration was significantly higher in the HOSG than in the LOSG, whereas the duration of CPAP administration was higher in the LOSG than in the HOSG. Invasive ventilation duration did not differ significantly between the groups. A significantly increased rate of IVH ≥ grade 2 in the HOSG is another interesting finding of our study. Previous evidence indicates that in at least a large proportion of cases, the IVH is preceded and possibly caused by a phase of low cerebral perfusion, leading to a reperfusion injury when the perfusion recovers (31). Higher SpO_2_ targets may aggravate low cerebral perfusion by lowering pulmonary vascular resistance and increasing left-to-right shunting of arterial blood through the still patent ductus arteriosus (PDA). In this case, early cerebral perfusion may have been worse in the HOSG, which may explain the increased IVH incidence. Only a fraction of the difference in the rate of IVH may be related to the higher rate of antenatal steroid prophylaxis in the LOSG. Antenatal steroid administration is known to reduce the risk of IVH in preterm infants (14, 32). Assuming an approximate 50% risk reduction for IVH by antenatal steroids (32) and an IVH incidence of 26.6%, as in the HOSG, a 15% difference in antenatal steroid use can only account for a 3% difference in the IVH incidence, whereas we found 8.9%. On the other hand, the difference in IVH between LOSG and HOSG may also be related to other differences in clinical procedures between different hospitals or even be a chance finding.

In a retrospective multicenter study, differences in applied treatments and results may reflect differences in clinical status or differences in the habits of caregivers. In the former case, such differences may strengthen our results, but they may become confounders in the latter. The higher use of inhaled and systemic steroids in the HOSG may be related to the higher incidence of BPD following more exposure to oxygen toxicity (33, 34). The frequent use of ibuprofen in the LOSG to constrict the ductus arteriosus may indicate a higher spontaneous closure rate in the HOSG (19, 35). Furthermore, the frequent use of ventilation with NO and HFOV in the LOSG may be related to a higher incidence of pulmonary hypertension when targeting lower oxygen saturations (36, 37).

We designed this study as a pragmatic approach, comparing management strategies rather than actual saturation values, which was similarly done in the NeOProM trials. However, our study has several limitations. We had to classify hospitals by their SpO_2_ target standards, which may introduce bias by other differences in treatment modalities into our analysis: as saturation targeting is only one aspect of care, all outcomes could be influenced by other treatment philosophies like the use of umbilical catheters, formula milk, and advancing milk feeds, as well as differences in baseline characteristics between our saturation groups like proportion of multiple birth and gestational age. We did not adjust any of the outcomes for these potential differences. At least we can exclude bias from not following the SpO_2_ target standards by checking how standards were followed in a randomly selected subsample of the patients of each hospital. The dataset is from 2008 to 2012 and therefore it is slightly aged, which, however, carries the advantage that treatments were not yet influenced by the NeOProM results. Furthermore, compared to the NeOProM trials with over 4,900 cases, we were only able to include 1,399 infants, which results in a lower statistical power. In addition, our number of patients is unevenly distributed among our groups, and the HOSG is smaller. In analyzing how centers followed their targets, we only used the saturation values from the 13–60 h of life, whereas NeOProM separated their saturation groups until 36 weeks postmenstrual age. Finally, multiple statistical tests carry the risk of getting significant results just by chance. We therefore preplanned the tests to be done and reported and refrained from fishing for differences.

Altogether, the results of the NeoProM trials cannot be transferred to all healthcare systems and locations: the five NeOProM trials observed no significant difference between a lower compared to a higher SpO_2_ target range on the “primary composite outcome of death or major disability at a corrected age of 18 to 24 months” (13), similar to our results, although we, unlike NeOProM, did not obtain separate counts on cerebral palsy, blindness, and deafness. We evaluated the Bayley cognitive or language scores: they were similar in lower and higher SpO_2_ target groups in NeOProM and in this study (13). As an MDI lower than 70 can indicate disability, our data also support this NeOProM result (23). Other outcomes were different. The rates of mortality and NEC were significantly higher in the lower SpO_2_ target groups on NeOProM, whereas we did not find a significant difference and not even a trend in this direction. ROP and BPD occurred much more frequently in the higher target groups of NeOProM, but no differences existed in this study. Similar results had previously been found in the STOP ROP trial regarding BPD (33). Summing up, results from trials could be influenced by one-site patient risks and local health hazards. Therefore, generalizations and transfers to other health systems should be made carefully. The rigorous design of randomized controlled blinded trials cannot eliminate all limitations of the NeOProM trials. Although the trials were planned to be consistent with uniform patient populations and SpO_2_ targets, some discrepancies in geography and methods may still have contributed to differences in outcome (38). For example, in the SUPPORT trial, unstable infants and those with pulmonary hypertension were not excluded (39), whereas COT excluded them (38). Hence, it is possible that there was an inclusion of more severely ill infants in SUPPORT (38). The exclusion of these infants in COT may have contributed to the vanished difference in mortality because a lower oxygen saturation may deteriorate pulmonary hypertension, which was only present in SUPPORT (37). Outborn infants, which may suffer from higher mortality and morbidity rates, were excluded from SUPPORT (38). BOOST-II UK enrolled a higher incidence of SGA infants than COT and SUPPORT trails (38), which could be one reason for the higher mortality and morbidity in the BOOST trials (38). Furthermore, the median oxygen saturations differed only by 2%–3% between the SpO_2_ target groups (10) because the lower SpO_2_ target group had higher than scheduled oxygen saturations (13, 17).

In COT, fewer “infants had median saturations below 85% or above 95%” than in SUPPORT, whereas the overlaps of the distributions of SpO_2_ between the two treatment groups in the range of 85% and 95% were larger in SUPPORT than in COT (17). These differences may also explain why COT did not find excess mortality in the low target group and excess retinopathy in the high target group (17).

In summary, although we cannot confirm or refute the NeOProM findings due to our study design, we did not observe that a lower SpO_2_ target range was an increased safety risk for extremely preterm infants in the area where this study was performed. We found an increased NEC rate at sites where higher SpO_2_ ranges were targeted. The rates of ROP and BPD, the mortality rate, and the MDI did not differ significantly between the SpO_2_ target groups. A significantly increased rate of IVH at sites where higher SpO_2_ ranges were targeted is an interesting aspect.

Thus, for the local health system, our study may indicate that the lower range posed fewer risks for preterm infants in the participating NICUs, as we did not see an increased number of deaths, BPD, NEC, IVH, or ROP in the lower SpO_2_ target group. As such, our data do not contradict the European consensus recommendations that saturations should be kept below 93% and never exceed 95% (40, 41). We cannot be sure that our outcome differences are associated with differences in oxygen saturations due to the retrospective study design and the discrepancies in baseline characteristics and site practices; the results should be interpreted cautiously. Further prospective studies might be desirable since we have seen that the NeOProM results cannot be easily generalized to other health systems.

## Data Availability

The data analyzed in this study is subject to the following licenses/restrictions: although the data are pseudonymized, reidentification of some cases cannot be ruled out completely. Therefore, the data cannot be made publicly available. Extracts can be obtained from the authors upon reasonable request. Requests to access these datasets should be directed to Nina.Willgerodt@medizin.uni-leipzig.de or Ulrich.Thome@medizin.uni-leipzig.de.

## Appendix

Diseases that led to exclusion from our data set as listed as follows (*n* = number of infants with this exclusion diagnosis, multiple mentions possible):

• Hypoplastic left heart syndrome, *n* = 2
• Hypoplastic right heart syndrome, *n* = 1
• Ventricular septal defect, *n* = 32
• Coarctation of the aorta, *n* = 2
• Truncus arteriosus communis, *n* = 2
• Tricuspid atresia, *n* = 1
• Other congenital malformation of the aorta arch, *n* = 1
• Hypoplastic aortic arch, *n* = 1
• Tetralogy of Fallot, *n* = 1
• Pulmonary valve stenosis, *n* = 10
• Pulmonary valve insufficiency, *n* = 3
• Pulmonary valve atresia, *n* = 1
• Stenosis of pulmonary artery, *n* = 7
• Transposition of the Great Arteries, *n* = 2
• High-grade mitral regurgitation, *n* = 6
• Esophageal atresia, *n* = 1
• Gastroschisis, *n* = 2
• Colon atresia, *n* = 1
• Small intestine atresia, *n* = 1
• Lung malformations (CAM, pulmonary dysplasia, tracheomalacia, congenital subglottic stenosis), *n* = 24
• Interstitial lung disease, *n* = 16
• 18 trisomy, *n* = 1
• Down's syndrome, *n* = 1
• Complex malformation syndrome, *n* = 1
• Congenital hydrocephalus, *n* = 5
• Omphalocele, *n* = 4
• Osteogenesis imperfecta, *n* = 3
• Hydrops fetalis, *n* = 1
• Diaphragmatic hernia, *n* = 1
