# Supplementary material for: Similar adverse outcome rates with high or low oxygen saturation targets in an area with low background mortality
Source: Front Pediatr. 2023 Oct 24;11:1235877. doi: 10.3389/fped.2023.1235877 (PMC10628636; doi:10.3389/fped.2023.1235877)
Supplement: Supplementary file 1 [file Table1.docx]

|  | **OR** | **95% -** | **CI** | **p-value** |
| --- | --- | --- | --- | --- |
|  |  | **Lower bound** | **Upper bound** |  |
| Cesarean section (compared to a spontaneous birth) | 0.80 | 0.48 | 1.36 | 0.42 |
| Gestational age (weeks) | 0.73 | 0.58 | 0.92 | <0.05 |
| Sex (female compared to male) | 0.58 | 0.40 | 0.83 | <0.05 |
| Age of mother (years) | 0.99 | 0.96 | 1.03 | 0.88 |
| Premature rupture of membranes | 1.35 | 0.91 | 2.02 | 0.14 |
| Chorioamnionitis | 0.89 | 0.54 | 1.47 | 0.65 |
| Birth weight (gram) | 0.99 | 0.99 | 0.99 | <0.05 |
| Apgar score at 5 minutes | 0.77 | 0.69 | 0.85 | <0.05 |
| Umbilical artery pH value | 0.96 | 0.62 | 1.48 | 0.85 |
| AGA (compared to SGA) | 0.59 | 0.32 | 1.13 | 0.11 |
| LGA (compared to SGA) | 0.62 | 0.06 | 6.84 | 0.70 |
| SpO_2_ target group (high compared to low SpO_2_ target group) | 1.30 | 0.88 | 1.91 | 0.18 |
| Maternal pre-eclampsia | 0.83 | 0.46 | 1.51 | 0.55 |
| Drug use of the mother****** | 0.91 | 0.47 | 1.76 | 0.78 |

**Binary logistic regression. The odds ratio (OR) is the ratio of the odds that an event will occur in a patient dying during the course to the odds that the event will occur in a patient not dying during the course. The results are presented with 95% confidence interval (CI) and two-sided p-value.**

**defined as consumption of nicotine, alcohol, or illegal drugs during pregnancy.
