# Supplementary material for: Similar adverse outcome rates with high or low oxygen saturation targets in an area with low background mortality
Source: Front Pediatr. 2023 Oct 24;11:1235877. doi: 10.3389/fped.2023.1235877 (PMC10628636; doi:10.3389/fped.2023.1235877)
Supplement: Supplementary file 2 [file Table2.docx]

|  | SUPPORT | | BOOST | | COT | | Our results | |
| --- | --- | --- | --- | --- | --- | --- | --- | --- |
|  | Lower SpO_2_ target | Higher SpO_2_ target | Lower SpO_2_ target | Higher SpO_2_ target | Lower SpO_2_ target | Higher SpO_2_ target | Lower SpO_2_ target | Higher SpO_2_ target |
| Death before discharge from hospital | 130/654 (19.9) * | 107/662 (16.2) * | 137/592 (23.1) * | 94/590 (15.9) * | 97/585 (16.6)^[[1]](#footnote-1)^ | 88/577 (15.3)^1^ | 130/1019 (12.8) | 56/380 (14.7) |
| Treated ROP before corrected age of 18-24 months | 41/475 (8.6)^4,^* | 91/509 (17.9)^4,^* | 58/479 (12.1) | 80/509 (15.7) | 64/500 (12.8)^[[2]](#footnote-2)^ | 66/503 (13.1)^2^ | 109/862 (12.6)^[[3]](#footnote-3)^ | 45/320 (14.1)^3^ |
| NEC | 76/641 (11.9) | 70/649 (10.8) | 72/592 (12.2) | 58/587 (9.9) | 74/602 (12.3) | 56/599 (9.3) | 52/1019 (5.1) * | 32/380 (8.4) * |
| Severe IVH | 83/630 (13.2) | 81/640 (12.7) | 74/579 (12.8) | 72/586 (12.3) | 123/598 (20.6)^[[4]](#footnote-4)^ | 136/589 (23.1)^4^ | 180/1019 (17.7)^[[5]](#footnote-5)^,* | 101/380 (26.6)^5^,* |
| Supplemental oxygen at postmenstrual age of 36 weeks | 203/540 (37.6) | 265/568 (46.7) | 201/456 (44.1) | 245/501 (48.9) | 164/515 (31.8)^[[6]](#footnote-6)^ | 171/517 (33.1)^6^ | 184/925 (19.9) | 85/338 (25.1) |

**Supplement, Comparison of the incidences in the NeOProM trials and our study.**

1. *Correlation is significant at the 0.05 level (2-tailed)

   defined as death before 18 months. [↑](#footnote-ref-1)
2. defined as severe ROP. [↑](#footnote-ref-2)
3. defined as ROP ≥ stage 3. [↑](#footnote-ref-3)
4. defined as brain injury, IVH included. [↑](#footnote-ref-4)
5. defined as IVH ≥ 2. [↑](#footnote-ref-5)
6. defined as severe BPD. [↑](#footnote-ref-6)
